# Identification of mutation of MYOC (c.1099G>A), a pedigree pathogenic gene of juvenile open angle glaucoma (JOAG): A case report

**DOI:** 10.1097/MD.0000000000040555

**Published:** 2024-11-22

**Authors:** Juan Zheng, Dongdong Zhao, Zhen Li, Changjun Feng, Zhaona Song, Rongrong Li, Bo Wang, Yaxin Liang, Xiufen Tian, Zhang Qianqian, Jianlu Gao

**Affiliations:** a Joint Laboratory for Translational Medicine Research, Liaocheng People’s Hospital, Liaocheng, Shandong, China; b Department of Ophthalmology, Liaocheng People’s Hospital, Liaocheng, Shandong, China; c Department of Clinical laboratory, Liaocheng People’s Hospital, Liaocheng, Shandong, China; d Department of Endodontics, Liaocheng People’s Hospital, Liaocheng, Shandong, China.

**Keywords:** JOAG, MYOC, pedigree pathogenic gene

## Abstract

**Rationale:**

The *MYOC* gene is associated with juvenile open-angle glaucoma (JOAG). This study aims to provide genetic counseling for a Chinese JOAG family by detecting MYOC mutations to identify high-risk individuals for early JOAG intervention. It also supplements the clinical characteristics of glaucoma patients with *MYOC* gene mutations.

**Patient concerns:**

A 43-year-old presented sought medical attention in a local hospital due to a 6-month decline in binocular vision. He was diagnosed as JOAG and underwent glaucoma surgery. The patient also had 11 family members with a history of JOAG.

**Diagnoses:**

After sequencing the polymerase chain reaction products of the patient, MYOC c.1099 G > A (p.G367R) mutation was observed. That is consistent with a diagnosis of JOAG.

**Intervention:**

Polymerase chain reaction analyses of 9 patients and 42 healthy family members were performed to explore potential mutations associated with familial JOAG.

**Outcomes:**

JOAG assisted in diagnosing the III-5 proband. Genetic detection indicated that III-5 was exposed to a novel heterozygous missense mutation of MYOC (c.1099 G > A [p.G367R]). The co-segregation of this gene with the trait observed in the pedigree was verified. All 10 participants exhibiting this mutation had JOAG phenotypes, whereas other participants did not show this mutation. In terms of MYOC mutation c.1099 G > A (p.G367R), this mutation occurred when the 1099th nucleotide in the encoding zone of MYOC changed from G to A. Moreover, the 367th amino acid coded by this base got mutated from glycine to arginine. DNAMAN sequence homology results showed that the G residues of MYOC: 367 were significantly conserved among different species. In addition, 3D protein conformation predicted that these mutations could decrease protein stability.

**Lessons:**

MYOC c.1099 G > A was identified as a pathogenic gene of JOAG in this pedigree. The addition of the MYOC mutant spectrum to JOAG in the Chinese population facilitates a complete understanding of the molecular pathogenesis and clinical diagnosis of MYOC.

## 1. Introduction

Glaucoma is the primary cause of irreversible visual impairment worldwide. By 2040, it is projected that the global population suffering from glaucoma will reach 111.8 million due to the growing number and proportion of elderly individuals.^[1]^ Primary open-angle glaucoma (POAG) is a common type of glaucoma that accounts for more than half of all glaucoma across European and American countries. In China, as the prevalence of myopia grows, there is also a corresponding increase in the proportion of glaucoma, especially POAG. Several factors affect POAG pathogenesis, and epidemiological studies have revealed that genetic and environmental factors can affect the development of POAG, with genetic factors being predominant.^[2]^ More than 20 POAG-related genes have been discovered. The primary causative genes of POAG consist of the glucocorticoid-induced response protein genes found in the trabecular meshwork, optic neuropathy-induced response protein genes, cytochrome genes, and tryptophan and aspartic acid repeat sequence 36 genes. Myocilin (*MYOC*) was the first pathogenic gene of POAG.^[3]^ Juvenile open-angle glaucoma (JOAG) is a specific form of POAG, characterized by an autosomal dominant inheritance pattern and typically appearing between the ages of 5 and 35. *MYOC* is associated with JOAG or early onset POAG, and approximately 1% to 4% of POAG is caused by this mutation. This mutation is primarily inherited dominantly and results from a missense mutation.^[4]^

POAG exhibits significant familial clustering, with positive family history being the primary risk factor. *MYOC* mutation is vital in POAG pathogenesis.^[5]^ Hence, genetic analysis on POAG patients and relatives has important clinical significance. This could aid in the early detection and treatment of the condition, thus averting permanent visual damage.

## 2. Experimental

The glaucoma pedigree originates from Yanggu County, Liaocheng, and pertains to individuals of Han ethnicity with no record of consanguineous marriage. A JOAG patient admitted to the ophthalmology department of Liaocheng People’s Hospital in March 2017 was the proband. Genetic traits were examined following a thorough examination of medical records and genetic analysis, resulting in the generation of a pedigree chart. Experienced ophthalmologists conducted thorough ocular evaluations on all available pedigree members, such as vision, intraocular pressure, slit-lamp, gonioscope, fundus, and visual field examination (some patients). This study adhered to the medical ethics and informed consent principles associated with studies involving human participants by the Helsinki Declaration and has been approved by the Ethics Committee of Liaocheng People’s Hospital (201706). A total of 4 mL of peripheral venous blood was collected from both JOAG pedigree patients and their relatives. The blood was collected using EDTA anticoagulant and then stored at −20°C.

The genomic DNA from venous blood was isolated, and then polymerase chain reaction primers were designed and synthesized to amplify the target region (Tables 1 and 2). Agarose gel electrophoresis was performed to detect whether the amplified product was the target gene, and then it was sequenced. The sequencing results were compared with the *MYOC* gene sequences retrieved from the NCBI GenBank database and analyzed with DNAMAN.

**Table 1:** Amplification primer sequence.

Exon	Primer sequence (forword/reverse)	Product size (bp)
MYOC1	PF5′-aaggccacccatccaggca′	766
MYOC1	PR5′-aagggcaggcagggagg′	
MYOC2	PF5′-ggtctcgaactcctgacctcag-3′	519
MYOC2	PR5′-ggctttgttagggaaaggctgac-3′	
MYOC3	PF5′-tactggctctgccaagcttcc-3′	978
MYOC3	PR5′-agcagtcaaagctgcctgg-3′	

**Table 2:** PCR reaction.

	Temperature (°C)	Time	Number of cycles
Pre-denaturation	95	10 min	1
Denaturation Annealing Extend	95	30 s	30
	63	30 s	
	72	2 min	
Total extension	72	10 min	1
Keep	4	–	–

PCR = polymerase chain reaction.

DNAMAN software was used to identify the mutation sites and to explore *MYOC* p.367G site conservation across different species. Swiss-Pdb Viewer (V4.10) was used to visualize the protein 3-dimensional (3D) structure of the mutant genes found in this screening. Finally, the impact of genetic variation on protein structure and function was assessed.

## 3. Results

### 3.1. Clinical phenotype

**Figure 1.**
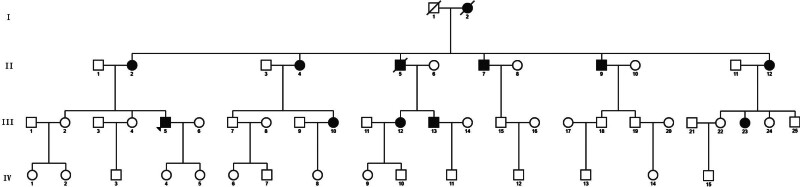
Genetic family diagram of JOAG. JOAG = juvenile open-angle glaucoma.

**Figure 2.**
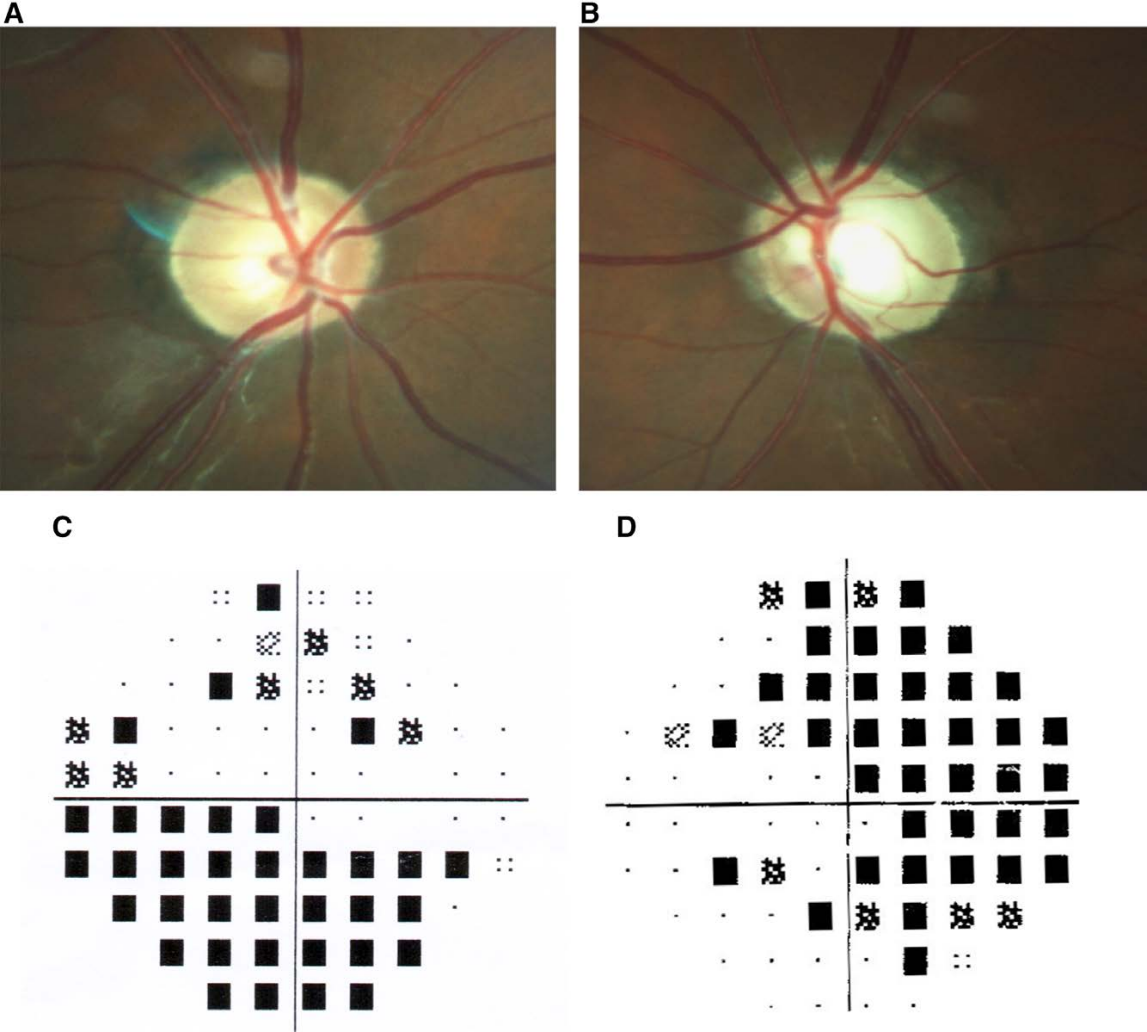
Fundus and visual field examination of the proband. (A) C/D 0.6 in the right eye of the proband. (B) C/D 0.8 in the left eye of the proband. (C) Patient with arcuate visual field defect in the right eye. (D) Patient with arcuate visual field defect in the left eye.

The study included a total of 54 family members spanning 4 generations in the pedigree. Out of the total, there were 12 individuals diagnosed with JOAG, consisting of 5 males and 7 females. JOAG patients were observed in 3 consecutive generations of this pedigree, and no history of consanguineous marriage was observed. JOAG was transmitted vertically from 1 generation to the next, with males and females suffering from the disease. JOAG was found not to be associated with gender and was consistent with autosomal dominant inheritance mode (Fig. 1). The proband, identified as III-5 and aged 31, sought medical attention at the Ophthalmology Department of Liaocheng People’s Hospital due to a 6-month decline in binocular vision. The ophthalmic examination revealed a best-corrected visual acuity of 0.8 in the right eye and 0.6 in the left eye. The intraocular pressure was measured to be 34 mm Hg in the right eye and 39 mm Hg in the left eye. The corneas in both eyes were clear, and the depth of the anterior chamber was satisfactory. Moreover, the crystalline lens exhibited transparency, and the boundary of the optic disc became clear and pale. The cup-to-disk ratio was enlarged by 0.6/0.8 (OD/OS), and the field of view revealed a defect in the double-arched vision field (Fig. 2). Therefore, the proband III-5 was diagnosed as JOAG. By analyzing the age at which glaucoma developed and conducting clinical examinations on patients with glaucoma in the family, it was determined that the average age of onset in this family was 27.7 ± 2.4 years. Additionally, all patients had undergone glaucoma surgery, but the management of intraocular pressure was ineffective, resulting in severe damage to the optic nerve (Table 3).

**Table 3:** Clinical information of glaucoma patients in the family.

Patient	Gender	Current age	Diagnosis age	Surgical method	Best corrected visual acuity	C/D
II-2	Female	72	31	Bilateral trabeculectomy	NLP/NLP	1.0/1.0
II-4	Female	64	24	Bilateral trabeculectomy	NLP/NLP	1.0/1.0
II-7	Male	61	30	Bilateral trabeculectomy	LP/LP	1.0/1.0
II-9	Male	57	26	Bilateral trabeculectomy	LP/NLP	1.0/1.0
II-12	Female	55	29	Bilateral trabeculectomy	0.4/0.1	0.9/1.0
III-5	Male	31	28	Bilateral trabeculectomy	0.8/0.6	0.6/0.8
III-10	Female	45	28	Bilateral trabeculectomy	1.0/0.9	0.9/0.9
III-12	Female	48	30	Bilateral trabeculectomy	0.3/0.2	0.9/1.0
III-13	Male	45	26	Bilateral trabeculectomy	0.5/1.0	0.9/0.9
III-23	Female	29	25	Bilateral trabeculectomy	0.4/0.2	0.9/0.9

### 3.2. 1.5% agarose gel electrophoresis of PCR products

**Figure 3.**
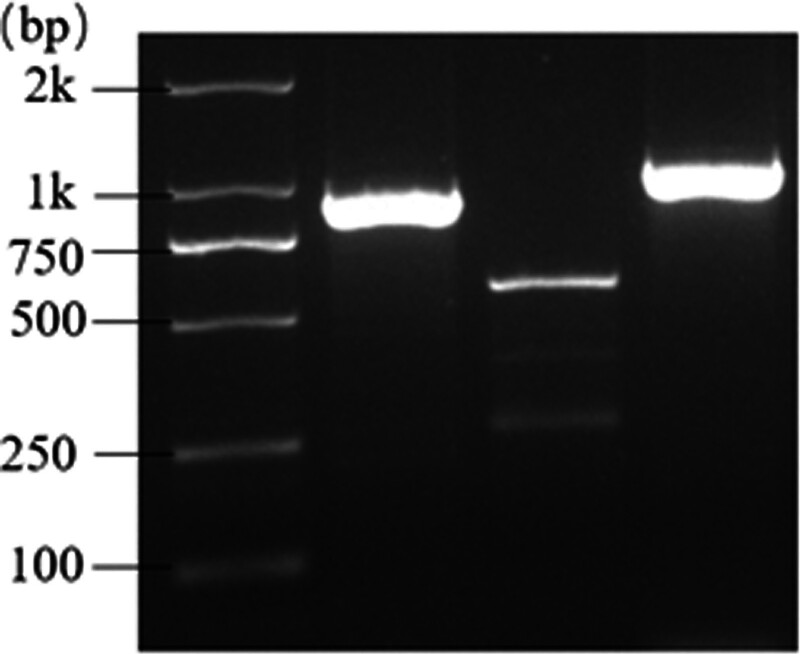
The agarose gel electrophoresis. PCR product fragments; Lane 1: *MYOC* exon 1, 766bp; Lane 2: *MYOC* exon 2, 519bp; Lane 3: *MYOC* exon 3, 987bp. PCR = polymerase chain reaction.

The primer sequences for the 3 exons of *MYOC* were designed and amplified based on the PCR cycle conditions. Agarose gel electrophoresis demonstrated that the 3 primer pairs successfully amplified the respective PCR products in the genomic DNA of each individual. The product had clear bands with high purity, without significant primer dimers or nonspecific bands, and had consistent size. All the PCR amplification products were specific DNA bands (Fig. 3).

### 3.3. Sequencing results

**Figure 4.**
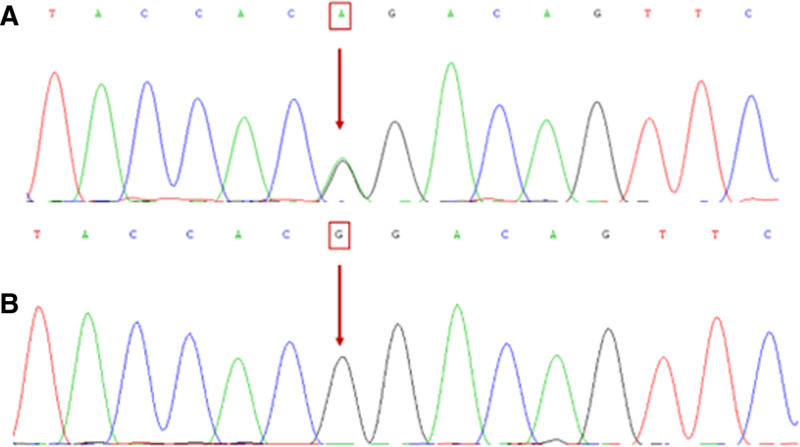
Identification of *MYOC* mutations. (A) Part of the *MYOC* sequence of proband III-5 (arrow indicates the presence of mutation). (B) Part of the *MYOC* sequence of healthy individuals III-7 (arrows indicate no mutation).

After sequencing the PCR products of 51 members, *MYOC* c.1099 G > A (p.G367R) polymorphism was observed in II: 2, 4, 7, 9, and 12, and III: 5, 10, 12, 13, and 24. Specifically, the 1099th base was identified in the encoding zone of *MYOC* and mutated from G to A, and the 367th amino acid encoded by this base mutated from glycine to arginine (Table 4 and Fig. 4). The remaining members of the family did not undergo any genetic mutations. The research results were consistent with phenotypic segregation (Table 5).

**Table 4:** Basic information of mutation sites.

Chr	Gene	Exon	Allele	AAChange.refGene	CytoBand
Chr1	*MYOC*	3	G > A	c.1099G > A:p.G367R	4q31.23

**Table 5:** Genotypes and phenotypes of family members.

Member	Genotype	Phenotype	Member	Genotype	Phenotype
II-1	GG	Normal	II-8	GG	Normal
II-2	GA	JOAG	II-9	GA	JOAG
II-3	GG	Normal	II-10	GG	Normal
II-4	GA	JOAG	II-11	GG	Normal
II-6	GG	Normal	II-12	GA	JOAG
II-7	GA	JOAG			
III-1	GG	Normal	III-14	GG	Normal
III-2	GG	Normal	III-15	GG	Normal
III-3	GG	Normal	III-16	GG	Normal
III-4	GG	Normal	III-17	GG	Normal
III-5	GA	JOAG	III-18	GG	Normal
III-6	GG	Normal	III-19	GG	Normal
III-7	GG	Normal	III-20	GG	Normal
III-8	GG	Normal	III-21	GG	Normal
III-9	GG	Normal	III-22	GG	Normal
III-10	GA	JOAG	III-23	GA	JOAG
III-11	GG	Normal	III-24	GG	Normal
III-12	GA	JOAG	III-25	GG	Normal
III-13	GA	JOAG			
IV-1	GG	Normal	IV-9	GG	Normal
IV-2	GG	Normal	IV-10	GG	Normal
IV-3	GG	Normal	IV-11	GG	Normal
IV-4	GG	Normal	IV-12	GG	Normal
IV-5	GG	Normal	IV-13	GG	Normal
IV-6	GG	Normal	IV-14	GG	Normal
IV-7	GG	Normal	IV-15	GG	Normal
IV-8	GG	Normal			

JOAG = juvenile open-angle glaucoma.

### 3.4. 3D structure

**Figure 5.**
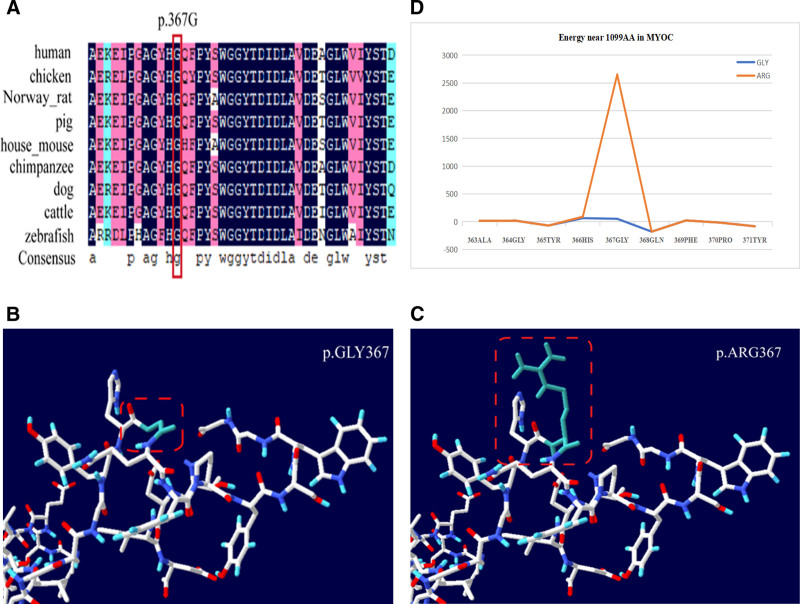
(A) *MYOC*: 367 G across species. (B–D) Schematic diagrams of the 3D protein structure of MYOC prior to and after the C > D mutation, which altered the amino acid at position 1099 from a glycine to an arginine. Changes in the force field proximal to this amino acid are shown. Boxes indicate the amino acid at this site before and after mutation. Blue lines represent the energy before this change in amino acid sequence, and red lines represent the energy after this change in amino acid sequence.

In terms of polymorphism of *MYOC* c.1099 G > A (p.G367R), the 1099th base within the encoding zone of *MYOC* mutated from G to A, and the 367th amino acid was encoded by this base and mutated from glycine to arginine. The amino acid sequence homology was assessed by comparing it using DNAMAN. The findings suggested that *MYOC*: 367 G showed evolutionary conservatism across several species. Moreover, the mutation of a conservative sequence revealed a significant impact (Fig. 5A). Amino acid mutations were visualized using the Swiss-Pdb Viewer (V4.10) to explore the potentially destructive nature of *MYOC*: 367 G mutation. The examination of the protein structure showed that the minimum potential energy increased from 51.59 to 2650.59 after the 367th amino acid underwent mutation. The examination of the protein structure and force field revealed that MYOC: 367 G had an impact on the force field. The protein using low potential energy had a stable structure (Fig. 5B–D). Additionally, the prediction results from I-Mutant 2.0 indicate that the *MYOC* p.G367R mutation decreases protein stability at the 2 mutation sites.

Ten family members carrying MYOC gene mutations are all JOAG patients, including 4 males and 6 females. The patients are diagnosed between 24 and 31 years with an average age of 27.7 years. Family members who do not carry the mutation have normal clinical examination. It can be seen that the MYOC gene mutation is the pathogenic gene of this JOAG family.

## 4. Discussion

The hereditary aspect of POAG and *MYOC*, the initially identified disease-causing gene, plays a crucial role in the development of glaucoma, which has been shown in this study. Myocilin is present in various human tissues. However, its concentration varies greatly, with the trabecular meshwork and ciliary body of the eye having larger concentrations than other tissues.^[6]^ *MYOC* is situated at the GLC1A site, which is in 1q21~31. This gene is composed of 3 exons and 2 introns, with introns separating the exons. The exons consist of 604, 126, and 782 base pairs, with untranslated sections located at the 5′ and 3′ ends. More than 180 mutations can be found in *MYOC*. Among them, nearly 70 mutation sites are related to the onset of POAG.^[7]^ Most pathogenic mutations cluster on the third exon, with fewer in the 1st and 2nd exons. Genetic mutations have also been observed in the promoter, intron 1, and intron 2. MYOC exhibits solubility in Triton, facilitating the synthesis of dimers. Additionally, it has the ability to interact with normal cellular constituents, leading to the formation of insoluble aggregates in Triton. These are involved in the normal physiological activities of cells. Variant MYOC combines with other proteins to form large insoluble aggregates, leading to tissue lysis or apoptosis. The variant *MYOC* gene alters the secondary structure of the protein, which in turn affects its function and induces POAG development.^[8]^ Further, a research study has observed that MYOC was expressed in the myelin sheath of peripheral nerves, and MYOC variation decreased the optic nerve’s tolerance to pressure.^[9]^

This study observed a novel mutation site (c.1099 G > A) of *MYOC* in a Chinese fourth-generation autosomal dominant JOAG family. The missense mutation resulted in the substitution of arginine with glycine (p.G367R) at codon 367, which was well conserved. Over 30 *MYOC* mutation sites have been observed in the POAG pedigree. The common sites include c.1021 T > C, c.1109 C > T, and c.898 G > Ac.1133 A > G.^[10–14]^ Moreover, this study confirmed the pathogenic role of c.1099 G > A mutation in Chinese Han POAG patient pedigree. This study has verified that the c.1099 G > A mutation has a role in causing glaucoma in Chinese Han POAG patients. The onset of glaucoma induced by this mutation occurs at an early age, and there is no significant difference between genders. The response to glaucoma filtering surgery was poor, but the reaction to trabeculectomy surgery was satisfactory. The long-term management of intraocular pressure was inadequate, and the risk of blindness in patients was higher. Thus, there is a significant need for timely diagnosis, prompt therapy, and consistent postoperative monitoring for glaucoma patients with G367R mutations.

In this study, pathogenic gene mutation sites associated with JOAG in the family were identified, which helps in determining glaucoma severity, excludes suspected glaucoma patients at an early stage, and alleviates the psychological burden caused by the disease. However, there are still some limitations: It cannot explain the diverse relationship between gene mutations and clinical phenotypes in families with the same pathogenic gene, where patients display different physical signs and varying degrees of disease. Although a 3-dimensional structural analysis of this gene has been conducted, the precise mechanism by which MYOC gene mutations cause disease remains unclear. Our next step is to investigate the detailed pathogenesis of JOAG caused by the MYOC gene mutation while focusing on gene function.

In the present study, the *MYOC* c.1099 G > A mutation was identified in an autosomal dominant JOAG family, and the patient’s characteristic phenotype was summarized. The results illustrated the initial impact of the G367R *MYOC* mutant. Additionally, the results indicated that genetic detection could be applied to high-risk populations, and pathogenic gene screening can help with genetic counseling, early diagnosis, and treatment of POAG patients or carriers.

## Acknowledgments

We thank the family for participating in this study and contributing to the literature.

## Author contributions

**Conceptualization:** Dongdong Zhao.

**Data curation:** Changjun Feng.

**Formal analysis:** Zhen Li.

**Investigation:** Zhang Qianqian.

**Methodology:** Xiufen Tian.

**Project administration:** Juan Zheng.

**Resources:** Rongrong Li.

**Software:** Zhaona Song.

**Supervision:** Juan Zheng, Jianlu Gao.

**Validation:** Bo Wang.

**Visualization:** Yaxin Liang.

**Writing – original draft:** Juan Zheng.

**Writing – review & editing:** Jianlu Gao.
